# Supplementary material for: A new Gaussian curvature of the image surface based variational model for haze or fog removal
Source: PLoS One. 2023 Mar 23;18(3):e0282568. doi: 10.1371/journal.pone.0282568 (PMC10035838; doi:10.1371/journal.pone.0282568)
Supplement: S1 File — (ZIP) [file pone.0282568.s001.zip › Matlab Codes/Single-Image-Haze-Removal -Using-DCP/Single Image Haze Removal Using Dark Channel Prior.pdf]

Single Image Haze Removal Using Dark Channel Prior

Kaiming He[1]
[1]Department of Information Engineering
The Chinese University of Hong Kong

Jian Sun[2]
[2]Microsoft Research Asia

Xiaoou Tang[1,3]
[3]Shenzhen Institute of Advanced Technology
Chinese Academy of Sciences

Abstract *In this paper, we propose a simple but effective image prior - dark channel prior to remove haze from a single input image. The dark channel prior is a kind of statistics of the haze-free outdoor images. It is based on a key observation - most local patches in haze-free outdoor images contain some pixels which have very low intensities in at least one color channel. Using this prior with the haze imaging model, we can directly estimate the thickness of the haze and recover a high quality haze-free image. Results on a variety of outdoor haze images demonstrate the power of the proposed prior. Moreover, a high quality depth map can also be obtained as a by-product of haze removal.*

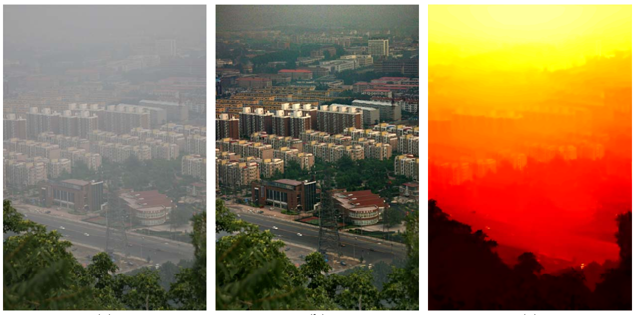

(a) (b) (c)

Figure 1. Haze removal using a single image. (a) input haze image. (b) image after haze removal by our approach. (c) our recovered depth map.

1. Introduction

Images of outdoor scenes are usually degraded by the turbid medium (*e.g.*, particles, water-droplets) in the atmosphere. Haze, fog, and smoke are such phenomena due to atmospheric absorption and scattering. The irradiance received by the camera from the scene point is attenuated along the line of sight. Furthermore, the incoming light is blended with the *airlight* [6] (ambient light reflected into the line of sight by atmospheric particles). The degraded images lose the contrast and color fidelity, as shown in Figure 1(a). Since the amount of scattering depends on the distances of the scene points from the camera, the degradation is spatial-variant.

Haze removal[1] (or dehazing) is highly desired in both consumer/computational photography and computer vision applications. First, removing haze can significantly increase the visibility of the scene and correct the color shift caused by the airlight. In general, the haze-free image is more visually pleasing. Second, most computer vision algorithms, from low-level image analysis to high-level object recognition, usually assume that the input image (after radiometric calibration) is the scene radiance. The performance of vision algorithms (*e.g.*, feature detection, filtering, and photometric analysis) will inevitably suffer from the biased, low-contrast scene radiance. Last, the haze removal can produce depth information and benefit many vision algorithms and advanced image editing. Haze or fog can be a useful depth clue for scene understanding. The bad haze image can be put to good use.

However, haze removal is a challenging problem because the haze is dependent on the unknown depth information. The problem is under-constrained if the input is only a single haze image. Therefore, many methods have been proposed by using multiple images or additional information. Polarization based methods [14, 15] remove the haze effect through two or more images taken with different degrees of polarization. In [8, 10, 12], more constraints are obtained from multiple images of the same scene under different weather conditions. Depth based methods [5, 11] require the rough depth information either from the user inputs or from known 3D models.

Recently, single image haze removal[2, 16] has made significant progresses. The success of these methods lies in using a stronger prior or assumption. Tan [16] observes that the haze-free image must have higher contrast compared with the input haze image and he removes the haze by maximizing the local contrast of the restored image. The results are visually compelling but may not be physically valid. Fattal [2] estimates the albedo of the scene and then infers the medium transmission, under the assumption that

[1]Haze, fog, and smoke differ mainly in the material, size, shape, and concentration of the atmospheric particles. See [9] for more details. In this paper, we do not distinguish these similar phenomena and use the term *haze removal* for simplicity.

978-1-4244-3991-1/09/$25.00 ©2009 IEEE 1956

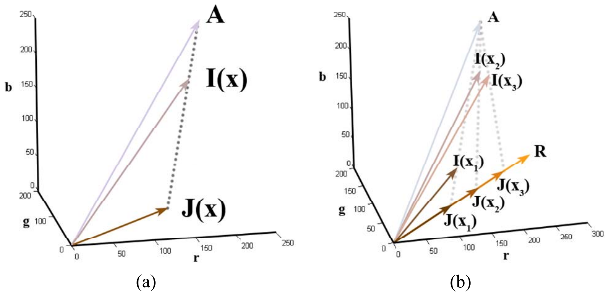

Figure 2. (a) Haze image formation model. (b) Constant albedo model used in Fattal's work [2].

the transmission and surface shading are locally uncorrelated. Fattal's approach is physically sound and can produce impressive results. However, this approach cannot well handle heavy haze images and may fail in the cases that the assumption is broken.

In this paper, we propose a novel prior - *dark channel prior*, for single image haze removal. The dark channel prior is based on the statistics of haze-free outdoor images. We find that, in most of the local regions which do not cover the sky, it is very often that some pixels (called "dark pixels") have very low intensity in at least one color (rgb) channel. In the haze image, the intensity of these dark pixels in that channel is mainly contributed by the airlight. Therefore, these dark pixels can directly provide accurate estimation of the haze's transmission. Combining a haze imaging model and a soft matting interpolation method, we can recover a hi-quality haze-free image and produce a good depth map (up to a scale).

Our approach is physically valid and are able to handle distant objects even in the heavy haze image. We do not rely on significant variance on transmission or surface shading in the input image. The result contains few halo artifacts.

Like any approach using a strong assumption, our approach also has its own limitation. The dark channel prior may be invalid when the scene object is inherently similar to the airlight over a large local region and no shadow is cast on the object. Although our approach works well for most of haze outdoor images, it may fail on some extreme cases. We believe that developing novel priors from different directions is very important and combining them together will further advance the state of the art.

2. Background

In computer vision and computer graphics, the model widely used to describe the formation of a haze image is as follows [16, 2, 8, 9]:

$$\mathbf{I}(\mathbf{x}) = \mathbf{J}(\mathbf{x})t(\mathbf{x}) + \mathbf{A}(1 - t(\mathbf{x})), \tag{1}$$

where $\mathbf{I}$ is the observed intensity, $\mathbf{J}$ is the scene radiance, $\mathbf{A}$ is the global atmospheric light, and t is the medium transmission describing the portion of the light that is not scattered and reaches the camera. The goal of haze removal is to recover $\mathbf{J}$, $\mathbf{A}$, and t from $\mathbf{I}$.

The first term $\mathbf{J}(\mathbf{x})t(\mathbf{x})$ on the right hand side of Equation (1) is called *direct attenuation* [16], and the second term $\mathbf{A}(1 - t(\mathbf{x}))$ is called *airlight* [6, 16]. Direct attenuation describes the scene radiance and its decay in the medium, while airlight results from previously scattered light and leads to the shift of the scene color. When the atmosphere is homogenous, the transmission t can be expressed as:

$$t(\mathbf{x}) = e^{-\beta d(\mathbf{x})}, \tag{2}$$

where β is the scattering coefficient of the atmosphere. It indicates that the scene radiance is attenuated exponentially with the scene depth d.

Geometrically, the haze imaging Equation (1) means that in RGB color space, vectors $\mathbf{A}$, $\mathbf{I}(\mathbf{x})$, and $\mathbf{J}(\mathbf{x})$ are coplanar and their end points are collinear (see Figure 2(a)). The transmission t is the ratio of two line segments:

$$t(\mathbf{x}) = \frac{\|\mathbf{A} - \mathbf{I}(\mathbf{x})\|}{\|\mathbf{A} - \mathbf{J}(\mathbf{x})\|} = \frac{A^c - I^c(\mathbf{x})}{A^c - J^c(\mathbf{x})}, \tag{3}$$

where $c \in \{r, g, b\}$ is color channel index.

Based on this model, Tan's method [16] focuses on enhancing the visibility of the image. For a patch with uniform transmission t, the visibility (sum of gradient) of the input image is reduced by the haze, since t<1:

$$\sum_{\mathbf{x}} \|\nabla \mathbf{I}(\mathbf{x})\| = t \sum_{\mathbf{x}} \|\nabla \mathbf{J}(\mathbf{x})\| < \sum_{\mathbf{x}} \|\nabla \mathbf{J}(\mathbf{x})\|. \tag{4}$$

The transmission t in a local patch is estimated by maximizing the visibility of the patch and satisfying a constraint that the intensity of $\mathbf{J}(\mathbf{x})$ is less than the intensity of $\mathbf{A}$. An MRF model is used to further regularize the result. This approach is able to unveil details and structures from the haze image. However, the output images tend to have larger saturation values because this method solely focuses on the enhancement of the visibility and does not intend to physically recover the scene radiance. Besides, the result may contain halo effects near the depth discontinuities.

In [2], Fattal proposes an approach based on Independent Component Analysis (ICA). First, the albedo of a local patch is assumed as a constant vector $\mathbf{R}$. Thus, all the $\mathbf{J}(\mathbf{x})$ in the patch have the same direction $\mathbf{R}$, as shown in Figure 2(b). Second, by assuming that the statistics of the surface shading $\|\mathbf{J}(\mathbf{x})\|$ and the transmission $t(\mathbf{x})$ are independent in the patch, the direction of $\mathbf{R}$ can be estimated by ICA. Finally, an MRF model guided by the input color image is applied to extrapolate the solution to the whole image. This approach is physics-based and can produce a

natural haze-free image together with a good depth map. However, because this approach is based on a statistically independent assumption in a local patch, it requires the independent components varying significantly. Any lack of variation or low signal-to-noise ratio (*e.g.*, in dense haze region) will make the statistics unreliable. Moreover, as the statistics is based on color information, it is invalid for grayscale images and difficult to handle dense haze which is often colorless and prone to noise.

In the next section, we will present a new prior - dark channel prior, to estimate the transmission directly from a hazy outdoor image.

3. Dark Channel Prior

The dark channel prior is based on the following observation on haze-free outdoor images: in most of the non-sky patches, at least one color channel has very low intensity at some pixels. In other words, the minimum intensity in such a patch should has a very low value. Formally, for an image $\mathbf{J}$, we define

$$J^{dark}(\mathbf{x}) = \min_{c \in \{r,g,b\}} \left(\min_{\mathbf{y} \in \Omega(\mathbf{x})} (J^c(\mathbf{y})) \right), \quad (5)$$

where J^c is a color channel of $\mathbf{J}$ and $\Omega(\mathbf{x})$ is a local patch centered at $\mathbf{x}$. Our observation says that except for the sky region, the intensity of J^{dark} is low and tends to be zero, if $\mathbf{J}$ is a haze-free outdoor image. We call J^{dark} the *dark channel* of $\mathbf{J}$, and we call the above statistical observation or knowledge the *dark channel prior*.

The low intensities in the dark channel are mainly due to three factors: a) shadows. *e.g.*, the shadows of cars, buildings and the inside of windows in cityscape images, or the shadows of leaves, trees and rocks in landscape images; b) colorful objects or surfaces. *e.g.*, any object (for example, green grass/tree/plant, red or yellow flower/leaf, and blue water surface) lacking color in any color channel will result in low values in the dark channel; c) dark objects or surfaces. *e.g.*, dark tree trunk and stone. As the natural outdoor images are usually full of shadows and colorful, the dark channels of these images are really dark!

To verify how good the dark channel prior is, we collect an outdoor image sets from flickr.com and several other image search engines using 150 most popular tags annotated by the flickr users. Since haze usually occurs in outdoor landscape and cityscape scenses, we manually pick out the haze-free landscape and cityscape ones from the downloaded images. Among them, we randomly select 5,000 images and manually cut out the sky regions. They are resized so that the maximum of width and height is 500 pixels and their dark channels are computed using a patch size 15×15. Figure 3 shows several outdoor images and the corresponding dark channels.

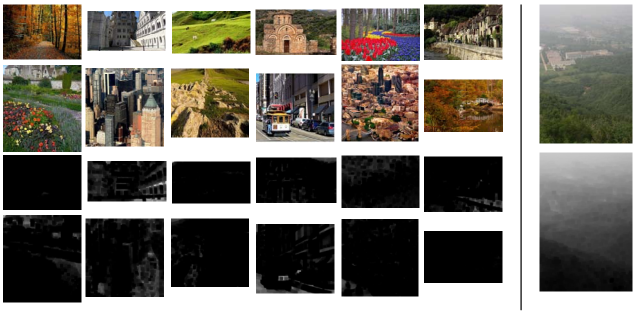

Figure 3. Top: example images in our haze-free image database. Bottom: the corresponding dark channels. Right: a haze image and its dark channel.

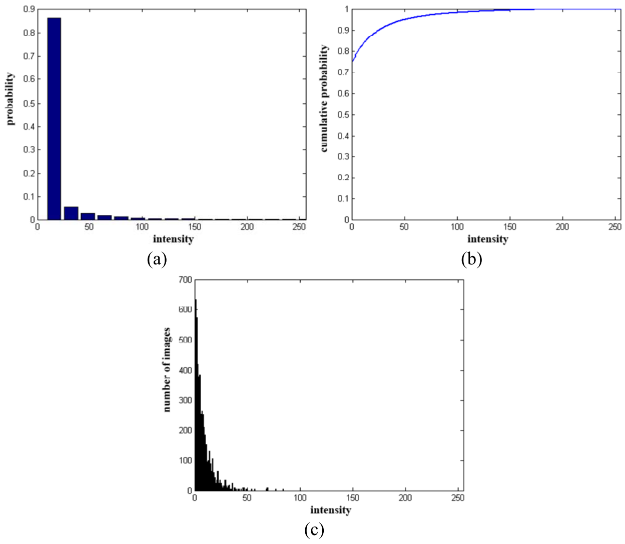

Figure 4. Statistics of the dark channels. (a) histogram of the intensity of the pixels in all of the 5,000 dark channels (each bin stands for 16 intensity levels). (b) corresponding cumulative distribution. (c) histogram of the average intensity of each dark channel.

Figure 4(a) is the intensity histogram over all 5,000 dark channels and Figure 4(b) is the corresponding cumulative histogram. We can see that about 75% of the pixels in the dark channels have zero values, and the intensities of 90% of the pixels are below 25. This statistic gives a very strong support to our dark channel prior. We also compute the average intensity of each dark channel and plot the corresponding histogram in Figure 4(c). Again, most dark channels have very low average intensity, meaning only a small portion of haze-free outdoor images deviate from our prior.

Due to the additive airlight, a haze image is brighter than its haze-free version in where the transmission t is low. So the dark channel of the haze image will have higher intensity in regions with denser haze. Visually, the intensity of the dark channel is a rough approximation of the thickness of the haze (see the right hand side of Figure 3). In the next

section, we will use this property to estimate the transmission and the atmospheric light.

Note that, we neglect the sky regions because the dark channel of a haze-free image may has high intensity here. Fortunately, we can gracefully handle the sky regions by using the haze imaging Equation (1) and our prior together. It is not necessary to cut out the sky regions explicitly. We discuss this issue in Section 4.1.

Our dark channel prior is partially inspired by the well known dark-object subtraction technique widely used in multi-spectral remote sensing systems. In [1], spatially homogeneous haze is removed by subtracting a constant value corresponding to the darkest object in the scene. Here, we generalize this idea and proposed a novel prior for natural image dehazing.

4. Haze Removal Using Dark Channel Prior

4.1. Estimating the Transmission

Here, we first assume that the atmospheric light $\mathbf{A}$ is given. We will present an automatic method to estimate the atmospheric light in Section 4.4. We further assume that the transmission in a local patch $\Omega(\mathbf{x})$ is constant. We denote the patch's transmission as $\tilde{t}(\mathbf{x})$. Taking the min operation in the local patch on the haze imaging Equation (1), we have:

$$\min_{\mathbf{y}\in\Omega(\mathbf{x})}(I^c(\mathbf{y})) = \tilde{t}(\mathbf{x}) \min_{\mathbf{y}\in\Omega(\mathbf{x})}(J^c(\mathbf{y})) + (1-\tilde{t}(\mathbf{x}))A^c. \quad (6)$$

Notice that the min operation is performed on three color channels independently. This equation is equivalent to:

$$\min_{\mathbf{y}\in\Omega(\mathbf{x})}(\frac{I^c(\mathbf{y})}{A^c}) = \tilde{t}(\mathbf{x}) \min_{\mathbf{y}\in\Omega(\mathbf{x})}(\frac{J^c(\mathbf{y})}{A^c}) + (1-\tilde{t}(\mathbf{x})). \quad (7)$$

Then, we take the min operation among three color channels on the above equation and obtain:

$$\min_c(\min_{\mathbf{y}\in\Omega(\mathbf{x})}(\frac{I^c(\mathbf{y})}{A^c})) = \tilde{t}(\mathbf{x}) \min_c(\min_{\mathbf{y}\in\Omega(\mathbf{x})}(\frac{J^c(\mathbf{y})}{A^c})) + (1-\tilde{t}(\mathbf{x})). \quad (8)$$

According to the dark channel prior, the dark channel J^{dark} of the haze-free radiance $\mathbf{J}$ tend to be zero:

$$J^{dark}(\mathbf{x}) = \min_c(\min_{\mathbf{y}\in\Omega(\mathbf{x})}(J^c(\mathbf{y}))) = 0. \quad (9)$$

As A^c is always positive, this leads to:

$$\min_c(\min_{\mathbf{y}\in\Omega(\mathbf{x})}(\frac{J^c(\mathbf{y})}{A^c})) = 0 \quad (10)$$

Putting Equation (10) into Equation (8), we can estimate the transmission $\tilde{t}$ simply by:

$$\tilde{t}(\mathbf{x}) = 1 - \min_c(\min_{\mathbf{y}\in\Omega(\mathbf{x})}(\frac{I^c(\mathbf{y})}{A^c})). \quad (11)$$

In fact, $\min_c(\min_{\mathbf{y}\in\Omega(\mathbf{x})}(\frac{I^c(\mathbf{y})}{A^c}))$ is the dark channel of the normalized haze image $\frac{I^c(\mathbf{y})}{A^c}$. It directly provides the estimation of the transmission.

As we mentioned before, the dark channel prior is not a good prior for the sky regions. Fortunately, the color of the sky is usually very similar to the atmospheric light $\mathbf{A}$ in a haze image and we have:

$$\min_c(\min_{\mathbf{y}\in\Omega(\mathbf{x})}(\frac{I^c(\mathbf{y})}{A^c})) \to 1, \text{ and } \tilde{t}(\mathbf{x}) \to 0,$$

in the sky regions. Since the sky is at infinite and tends to has zero transmission, the Equation (11) gracefully handles both sky regions and non-sky regions. We do not need to separate the sky regions beforehand.

In practice, even in clear days the atmosphere is not absolutely free of any particle. So, the haze still exists when we look at distant objects. Moreover, the presence of haze is a fundamental cue for human to perceive depth [3, 13]. This phenomenon is called *aerial perspective*. If we remove the haze thoroughly, the image may seem unnatural and the feeling of depth may be lost. So we can optionally keep a very small amount of haze for the distant objects by introducing a constant parameter ω ($0<\omega\leq1$) into Equation (11):

$$\tilde{t}(\mathbf{x}) = 1 - \omega \min_c(\min_{\mathbf{y}\in\Omega(\mathbf{x})}(\frac{I^c(\mathbf{y})}{A^c})). \quad (12)$$

The nice property of this modification is that we adaptively keep more haze for the distant objects. The value of ω is application-based. We fix it to 0.95 for all results reported in this paper.

Figure 5(b) is the estimated transmission map from an input haze image (Figure 5(a)) using the patch size 15×15. It is reasonably good but contains some block effects since the transmission is not always constant in a patch. In the next subsection, we refine this map using a soft matting method.

4.2. Soft Matting

We notice that the haze imaging Equation (1) has a similar form with the image matting equation. A transmission map is exactly an alpha map. Therefore, we apply a soft matting algorithm [7] to refine the transmission.

Denote the refined transmission map by $t(\mathbf{x})$. Rewriting $t(\mathbf{x})$ and $\tilde{t}(\mathbf{x})$ in their vector form as $\mathbf{t}$ and $\tilde{\mathbf{t}}$, we minimize the following cost function:

$$E(\mathbf{t}) = \mathbf{t}^T \mathrm{L} \mathbf{t} + \lambda(\mathbf{t} - \tilde{\mathbf{t}})^T(\mathbf{t} - \tilde{\mathbf{t}}). \quad (13)$$

where L is the Matting Laplacian matrix proposed by Levin [7], and λ is a regularization parameter. The first term is the smooth term and the second term is the data term.

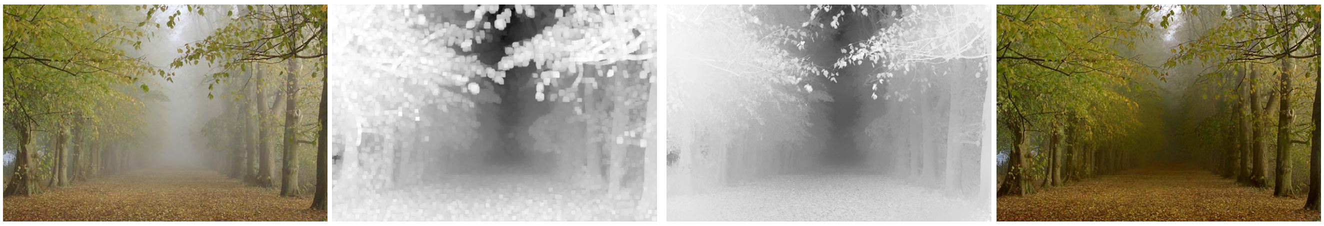
(a) (b) (c) (d)

Figure 5. Haze removal result. (a) input haze image. (b) estimated transmission map. (c) refined transmission map after soft matting. (d) final haze-free image.

The (i,j) element of the matrix L is defined as:

$$\sum_{k|(i,j)\in w_k} (\delta_{ij} - \frac{1}{|w_k|}(1+(\mathbf{I}_i-\mu_k)^T(\Sigma_k + \frac{\varepsilon}{|w_k|}\mathrm{U}_3)^{-1}(\mathbf{I}_j-\mu_k))), \tag{14}$$

where $\mathbf{I}_i$ and $\mathbf{I}_j$ are the colors of the input image $\mathbf{I}$ at pixels i and j, δ_{ij} is the Kronecker delta, μ_k and Σ_k are the mean and covariance matrix of the colors in window w_k, U_3 is a 3×3 identity matrix, ε is a regularizing parameter, and $|w_k|$ is the number of pixels in the window w_k.

The optimal $\mathbf{t}$ can be obtained by solving the following sparse linear system:

$$(\mathrm{L} + \lambda\mathrm{U})\mathbf{t} = \lambda\tilde{\mathbf{t}} \tag{15}$$

where U is an identity matrix of the same size as L. Here, we set a small value on λ (10^{-4} in our experiments) so that $\mathbf{t}$ is softly constrained by $\tilde{\mathbf{t}}$.

Levin's soft matting method has also been applied by Hsu *et al.* [4] to deal with the spatially variant white balance problem. In both Levin's and Hsu's works, the $\tilde{\mathbf{t}}$ is only known in sparse regions and the matting is mainly used to extrapolate the value into the unknown region. In this paper, we use the soft matting to refine a coarser $\tilde{\mathbf{t}}$ which has already filled the whole image.

Figure 5(c) is the soft matting result using Figure 5(b) as the data term. As we can see, the refined transmission map manages to capture the sharp edge discontinuities and outline the profile of the objects.

4.3. Recovering the Scene Radiance

With the transmission map, we can recover the scene radiance according to Equation (1). But the direct attenuation term $\mathbf{J}(\mathbf{x})t(\mathbf{x})$ can be very close to zero when the transmission $t(\mathbf{x})$ is close to zero. The directly recovered scene radiance $\mathbf{J}$ is prone to noise. Therefore, we restrict the transmission $t(\mathbf{x})$ to a lower bound t_0, which means that a small amount of haze are preserved in very dense haze regions. The final scene radiance $\mathbf{J}(\mathbf{x})$ is recovered by:

$$\mathbf{J}(\mathbf{x}) = \frac{\mathbf{I}(\mathbf{x}) - \mathbf{A}}{\max(t(\mathbf{x}), t_0)} + \mathbf{A}. \tag{16}$$

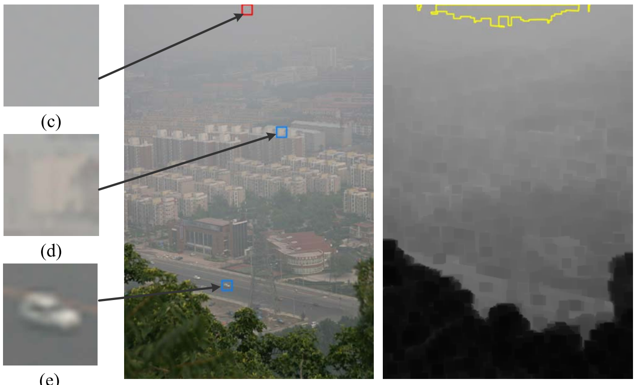
(c) (d) (e) (a) (b)

Figure 6. Estimating the atmospheric light. (a) input image. (b) dark channel and the most haze-opaque region. (c) patch from where our method automatically obtains the atmospheric light. (d) and (e): two patches that contain pixels brighter than the atmospheric light.

A typical value of t_0 is 0.1.Since the scene radiance is usually not as bright as the atmospheric light, the image after haze removal looks dim. So, we increase the exposure of $\mathbf{J}(\mathbf{x})$ for display. Figure 5(d) is our final recovered scene radiance.

4.4. Estimating the Atmospheric Light

In most of the previous single image methods, the atmospheric light $\mathbf{A}$ is estimated from the most haze-opaque pixel. For example, the pixel with highest intensity is used as the atmospheric light in [16] and is furthered refined in [2]. But in real images, the brightest pixel could be on a white car or a white building.

As we discussed in Section 3, the dark channel of a haze image approximates the haze denseness well (see Figure 6(b)). We can use the dark channel to improve the atmospheric light estimation. We first pick the top 0.1% brightest pixels in the dark channel. These pixels are most haze-opaque (bounded by yellow lines in Figure 6(b)). Among these pixels, the pixels with highest intensity in the input image $\mathbf{I}$ is selected as the atmospheric light. These pixels

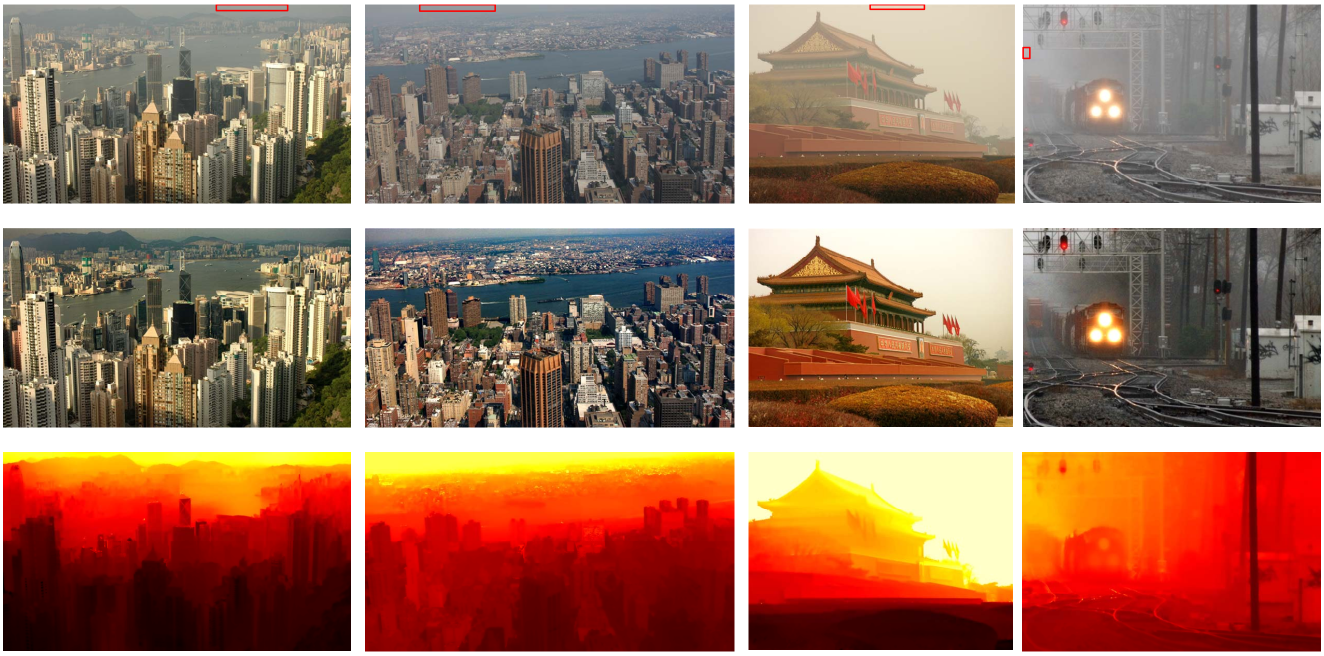

Figure 7. Haze removal results. Top: input haze images. Middle: restored haze-free images. Bottom: depth maps. The red rectangles in the top row indicate where our method automatically obtains the atmospheric light.

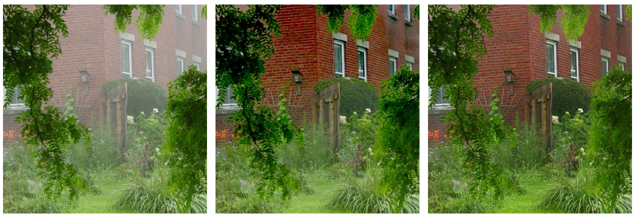

Figure 9. Comparison with Fattal's work [2]. Left: input image. Middle: Fattal's result. Right: our result.

are in the red rectangle in Figure 6(a). Note that these pixels may not be brightest in the whole image.

This simple method based on the dark channel prior is more robust than the "brightest pixel" method. We use it to automatically estimate the atmospheric lights for all images shown in the paper.

5. Experimental Results

In our experiments, we perform the local min operator using Marcel van Herk's fast algorithm [17] whose complexity is linear to image size. The patch size is set to 15×15 for a 600×400 image. In the soft matting, we use Preconditioned Conjugate Gradient (PCG) algorithm as our solver. It takes about 10-20 seconds to process a 600×400 pixel image on a PC with a 3.0 GHz Intel Pentium 4 Processor.

Figure 1 and Figure 7 show our haze removal results and the recovered depth maps. The depth maps are computed using Equation (2) and are up to an unknown scaling parameter β. The atmospheric lights in these images are automatically estimated using the method described in Section 4.4. As can be seen, our approach can unveil the details and recover vivid color information even in very dense haze regions. The estimated depth maps are sharp and consistent with the input images.

In Figure 8, we compare our approach with Tan's work [16]. The colors of his result are often over saturated, since his algorithm is not physically based and may underestimate the transmission. Our method recovers the structures without sacrificing the fidelity of the colors (*e.g.*, swan). The halo artifacts are also significantly small in our result.

Next, we compare our approach with Fattal's work. In Figure 9, our result is comparable to Fattal's result [2]. In Figure 10, we show that our approach outperforms Fattal's in situations of dense haze. Fattal's method is based on statistics and requires sufficient color information and variance. If the haze is dense, the color is faint and the variance is not high enough for his method to reliably estimate the transmission. Figure 10 (b) and (c) show his results before and after the MRF extrapolation. Since only parts of transmission can be reliably recovered, even after extrapolation, some of these regions are too dark (mountains) and some hazes are not removed (distant part of the cityscape). On the contrary, our approach works well for both regions

[2] from http://www.cs.huji.ac.il/~raananf/projects/defog/index.html

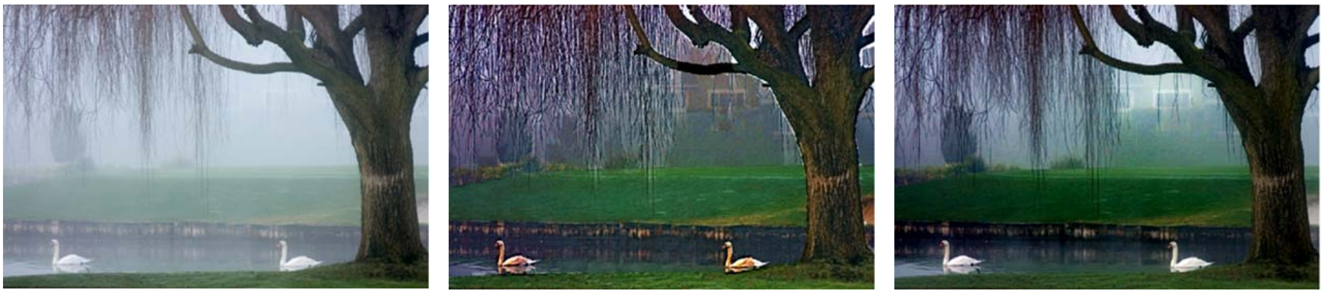

Figure 8. Comparison with Tan's work [16]. Left: input image. Middle: Tan's result. Right: our result.

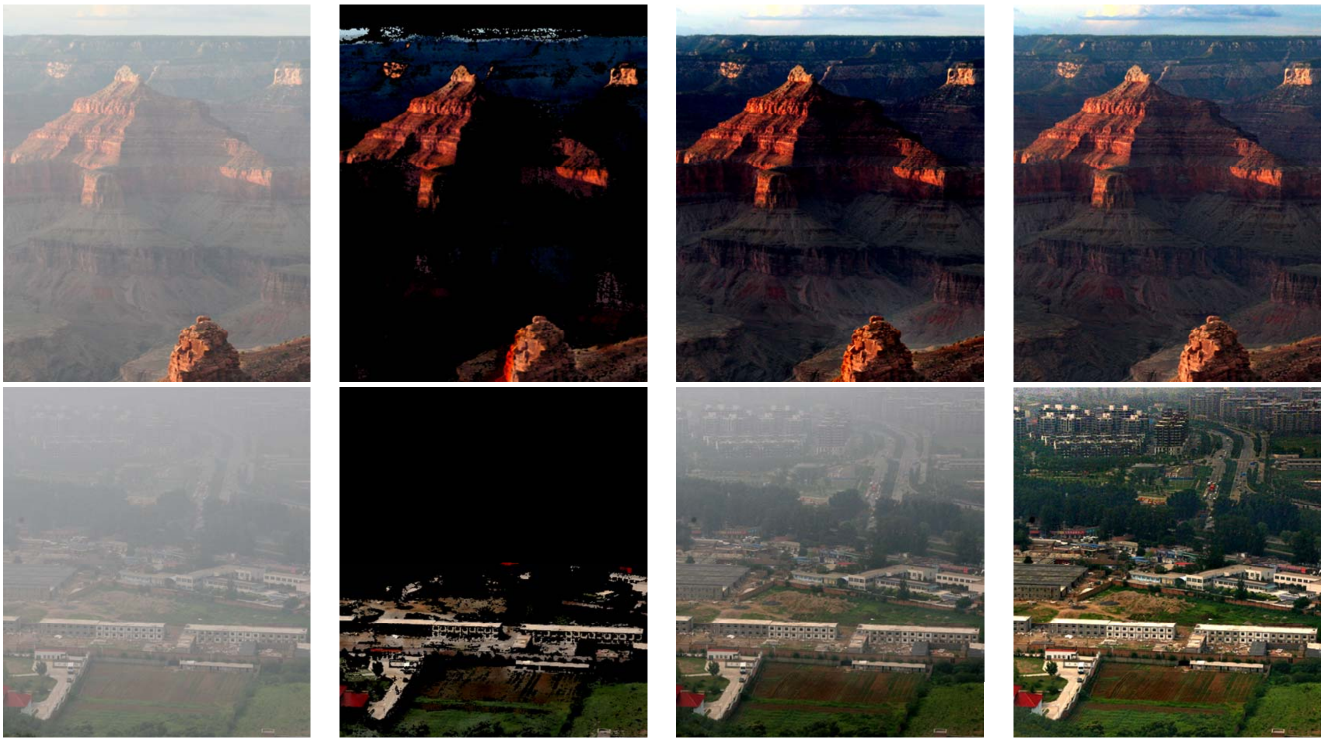

(a) (b) (c) (d)

Figure 10. More comparisons with Fattal's work [2]. (a) input images. (b) results before extrapolation, using Fattal's method. The transmission is not estimated in the black regions. (c) Fattal's results after extrapolation. (d) our results.

(Figure 10(d)).

We also compare our method with Kopf *et al.*'s very recent work [5] in Figure 11. To remove the haze, they utilize the 3D models and texture maps of the scene. This additional information may come from Google Earth and satellite images. However, our technique can generate comparable results from a single image without any geometric information.

Our approach even works for the gray-scale images if there are enough shadow regions in the image. We omit the operator $\min_c$ and use the gray-scale form of soft matting [7]. Figure 12 shows an example.

[More results and comparisons can be found at http://mmlab.ie.cuhk.edu.hk]

6. Discussions and Conclusions

In this paper, we have proposed a very simple but powerful prior, called dark channel prior, for single image haze removal. The dark channel prior is based on the statistics of the outdoor images. Applying the prior into the haze imaging model, single image haze removal becomes simpler and more effective.

Since the dark channel prior is a kind of statistic, it may not work for some particular images. When the scene objects are inherently similar to the atmospheric light and no

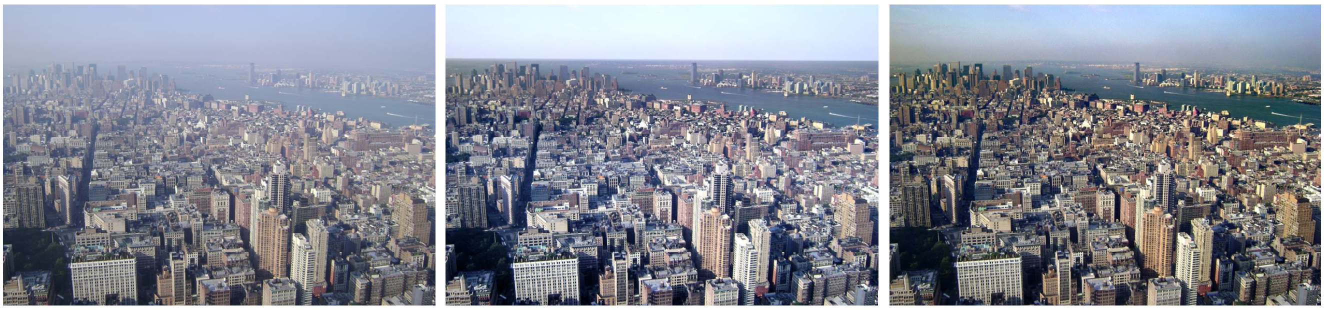

Figure 11. Comparison with Kopf *et al.*'s work [5]. Left: input image. Middle: Kopf's result. Right: our result.

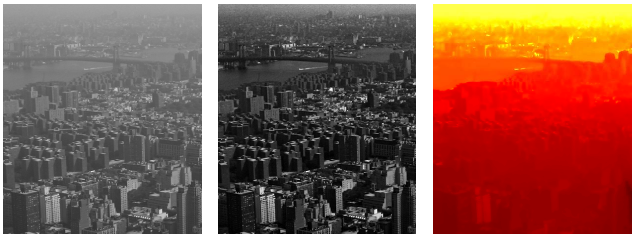

Figure 12. Gray-scale image example.

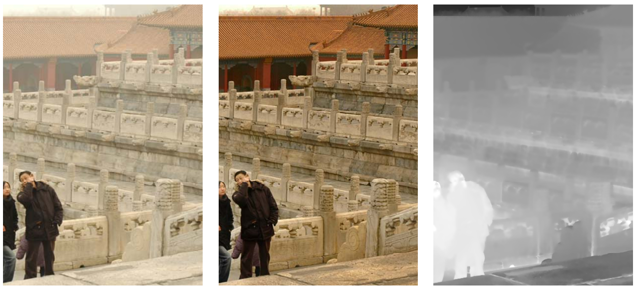

Figure 13. Failure case. Left: input image. Middle: our result. Right: our transmission map. The transmission of the marble is underestimated.

shadow is cast on them, the dark channel prior is invalid. Our method will underestimate the transmission for these objects, such as the white marble in Figure 13.

Our work also shares the common limitation of most haze removal methods - the haze imaging model may be invalid. More advanced models [13] can be used to describe complicated phenomena, such as the sun's influence on the sky region, and the blueish hue near the horizon. We intend to investigate haze removal based on these models in the future.

References

[1] P. Chavez. An improved dark-object substraction technique for atmospheric scattering correction of multispectral data. *Remote Sensing of Environment*, 24:450–479, 1988. 4

[2] R. Fattal. Single image dehazing. In *SIGGRAPH*, pages 1–9, 2008. 1, 2, 5, 6, 7

[3] E. B. Goldstein. Sensation and perception. 1980. 4

[4] E. Hsu, T. Mertens, S. Paris, S. Avidan, and F. Durand. Light mixture estimation for spatially varying white balance. In *SIGGRAPH*, pages 1–7, 2008. 5

[5] J. Kopf, B. Neubert, B. Chen, M. Cohen, D. Cohen-Or, O. Deussen, M. Uyttendaele, and D. Lischinski. Deep photo: Model-based photograph enhancement and viewing. *SIGGRAPH Asia*, 2008. 1, 7, 8

[6] H. Koschmieder. Theorie der horizontalen sichtweite. *Beitr. Phys. Freien Atm.*, 12:171–181, 1924. 1, 2

[7] A. Levin, D. Lischinski, and Y. Weiss. A closed form solution to natural image matting. *CVPR*, 1:61–68, 2006. 4, 7

[8] S. G. Narasimhan and S. K. Nayar. Chromatic framework for vision in bad weather. *CVPR*, pages 598–605, 2000. 1, 2

[9] S. G. Narasimhan and S. K. Nayar. Vision and the atmosphere. *IJCV*, 48:233–254, 2002. 1, 2

[10] S. G. Narasimhan and S. K. Nayar. Contrast restoration of weather degraded images. *PAMI*, 25:713–724, 2003. 1

[11] S. G. Narasimhan and S. K. Nayar. Interactive deweathering of an image using physical models. In *Workshop on Color and Photometric Methods in Computer Vision*, 2003. 1

[12] S. K. Nayar and S. G. Narasimhan. Vision in bad weather. *ICCV*, page 820, 1999. 1

[13] A. J. Preetham, P. Shirley, and B. Smits. A practical analytic model for daylight. In *SIGGRAPH*, pages 91–100, 1999. 4, 8

[14] Y. Y. Schechner, S. G. Narasimhan, and S. K. Nayar. Instant dehazing of images using polarization. *CVPR*, 1:325, 2001. 1

[15] S. Shwartz, E. Namer, and Y. Y. Schechner. Blind haze separation. *CVPR*, 2:1984–1991, 2006. 1

[16] R. Tan. Visibility in bad weather from a single image. *CVPR*, 2008. 1, 2, 5, 6, 7

[17] M. van Herk. A fast algorithm for local minimum and maximum filters on rectangular and octagonal kernels. *Pattern Recogn. Lett.*, 13:517–521, 1992. 6
