# Supplementary figures and images for: A new Gaussian curvature of the image surface based variational model for haze or fog removal
Source: PLoS One. 2023 Mar 23;18(3):e0282568. doi: 10.1371/journal.pone.0282568 (PMC10035838; doi:10.1371/journal.pone.0282568)

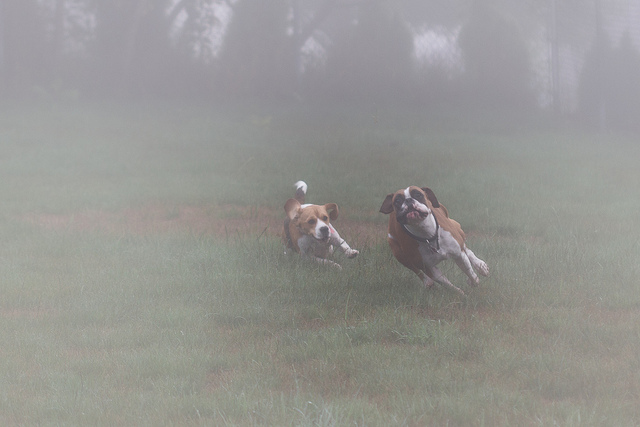

Supplement: S1 File — (ZIP) [file pone.0282568.s001.zip › Matlab Codes/Single-Image-defogging-method/Dogs.png]

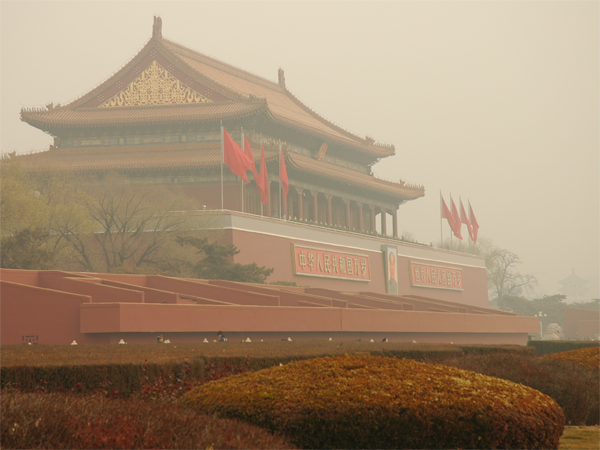

Supplement: S1 File — (ZIP) [file pone.0282568.s001.zip › Matlab Codes/Single-Image-defogging-method/Palace.png]

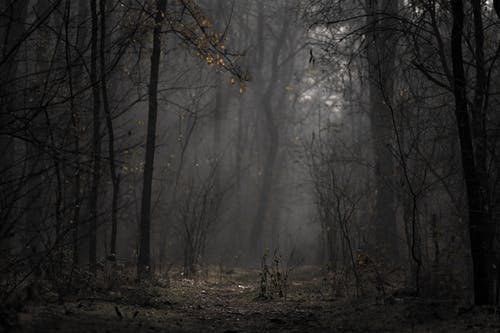

Supplement: S1 File — (ZIP) [file pone.0282568.s001.zip › Matlab Codes/Single-Image-defogging-method/Forest.jpeg]

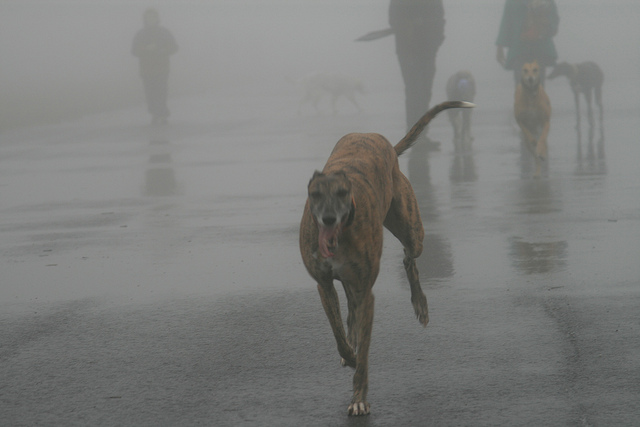

Supplement: S1 File — (ZIP) [file pone.0282568.s001.zip › Matlab Codes/Single-Image-defogging-method/Dog.png]

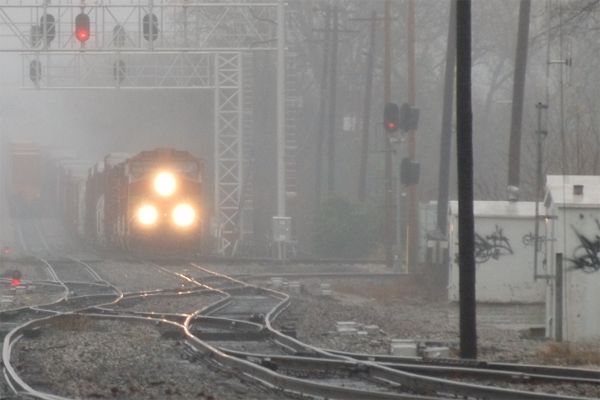

Supplement: S1 File — (ZIP) [file pone.0282568.s001.zip › Matlab Codes/Single-Image-defogging-method/Train.png]
