# Supplementary material for: A new Gaussian curvature of the image surface based variational model for haze or fog removal
Source: PLoS One. 2023 Mar 23;18(3):e0282568. doi: 10.1371/journal.pone.0282568 (PMC10035838; doi:10.1371/journal.pone.0282568)
Supplement: Attachment — Submitted filename: Reply_PLOS_one_D_22_17575_R1.pdf [file pone.0282568.s002.pdf]

Reply: A New Gaussian Curvature of the Image Surface Based Variational Model for Haze or Fog Removal

Muhammad Arif[1], Noor Badshah[2], Tufail Ahmad Khan[3], and Asmat Ullah[4]

[1]University of Engineering and Technology, Peshawar, Pakistan. E-mail: rfktk1@gmail.com.
[2]University of Engineering and Technology, Peshawar, Pakistan. E-mail: noorbadshah@uet.edu.pk.
[3]University of Engineering and Technology, Peshawar, Pakistan. E-mail: tufailmarwat@uetpeshawar.edu.pk.
[4]University of Engineering and Technology, Peshawar, Pakistan. E-mail: asmatullah75@gmail.com, asmatullah75@outlook.com.

Ms. Ref. No.: PONE-D-22-17575

Dear sir,

Thank you for your useful comments and suggestions on the language, mathematical mistakes and structure of our manuscript. We have modified the manuscript accordingly, and detailed corrections are listed below point by point.

First Reviewer:

- The contribution and motivation is not clearly summarized.
  Reply:-We have revised the contribution part of the paper according to your valuable comment/s. For more details please see the revise version of the manuscript (especially see page No. 3 and 4 of Section 1). Hope our revise work will be given due consideration.

- What is the main difference of the presented M3 comparing to M1 and M2.
  Reply:-M1: uses the variational approach with edge-preserving regularization for estimation of air-light map. By using the fundamental lemma of calculus of variations, the Euler Lagrange equation was derived for atmospheric veil that can find the maxima of a given functional. This equation was solved by using a gradient decent method and time parameter. Then, the estimated atmospheric veil was obtained, and then have conducted the image restoration by using inferred atmospheric veil. Finally, to improve the contrast of restoration image various histogram equalization methods were employed.

  M2: proposed a simple and effective image priordark channel prior to remove haze from a single input image. The dark channel prior is a kind of statistics of outdoor haze-free images. It is based on a key observationmost local patches in outdoor haze-free images contain some pixels whose intensity is very low in at least one color channel. Using this prior with the haze imaging model, M2 can directly estimate the thickness of the haze and recover a good quality haze-free image. Results on a variety of hazy images demonstrate the power of the proposed prior. Moreover, a high-quality depth map can also be obtained as a byproduct of haze removal. M2 used the soft matting algorithm to annihilate halo artifacts generated by the kernel operation and refined the transmission map. They improved the de-hazing results well but the soft matting algorithm was very costly and time

consuming.

M3: propose a new model based on dark channel prior and using Gaussian curvature (GC) as a regularizer (Lee et al. [30] stated that only Gaussian curvature can maintain important structures (such as corners and wrinkles). The work was supported by Elsey and Esedoglu [28] and proved that the Gaussian curvature of the surface is a good preserver for important features of image as compared to mean curvature.). Dark channel prior depends on key perception-most local patches in haze/fog-free (outdoor) images contain a few pixels which have extremely low intensities in at least one color channel. Utilizing this prior to the haze/fog imaging model, we can straightforwardly approximate the thickness of the haze and recuperate a good-quality haze/fog-free image. This model uses advantages of both DCP and GC, which leads to good restoration results, because dark channel prior performs well for estimating the thickness of the haze/fog and recovering good quality scene, while Gaussian curvature performs well for eliminating staircase effect, preserving textures and sharp edges. The resulting model has the advantage of better preserving image regions containing textures and fine details, leading to a more natural scene while reducing the staircase effect in smooth regions. Hence motivated by the advantages of Gaussian curvature compared to the mean curvature and total variation in 2D image de-noising pointed out by Elsey and Esedoglu [28] and Lee and Seo [30] in geometry processing and dark channel prior, here we design a Gaussian curvature of the scene surface regularization and DCP based model for single image de-hazing. Hence employing this model, reasonable improvement in image quality is obtained.

It is worth mentioning to state that the proposed model (M3) combined the Dark Channel Prior with Gaussian curvature for the first time to image de-hazing/de-fogging phenomena. The augmented Lagrangian method is used to find the minimizer of the proposed functional, which will be a system of partial differential equations. To get fast convergence, fast Fourier transforms (FFT) is used to solve the system of PDEs. The performance of the proposed model is compared with other state-of-the-art models qualitatively and quantitatively. The proposed model is tested on various real and synthetic images which show better efficiency in staircase effects reduction, haze/fog removal, image contrast, corners, and sharp edges conservation respectively.

As there are many types of hazy or foggy images captured under different homogeneous and inhomogeneous weather conditions like Outdoor and Indoor Scenes, Synthetic Road Scene Images Under Heterogeneous Fog, Realistic CCTV Camera Captured Images Under Heterogeneous Haze etc. In this paper, we have performed experiments on large number of different type images in order to validate the performance of the proposed model and its authenticity and to endorse the proposed model performance, we have included the famous Real-World Foggy Image Data-set (MRFID) as well. The test/s qualitative and quantitative results have demonstrated that the M3-method is more effective while preserving sharp edges, contrast and other image details during the haze or fog removal process quite well than other comparing methods. For more details, please see the revise version of the manuscript.

Applications: The applications of the proposed method may be extended to cover image segmentation, image inpainting, inland river image processing, road scenes image processing under homogeneous and heterogeneous haze or fog, defense and surveillance images, underwater image processing, and hazy or foggy videos processing.

- Algorithm 1 is given based on Eqs 21-26. However it is difficult to reproduce the results. Please note that the first step we need to know the value of A to determine q, while we further need the pre-specified q to obtain A. In my opinion, there are coupled to be calculated. Please give the true pseudo code to make the algorithm reproducible.

  Reply:- We have provided the related pseudo MATLAB code of the proposed method for your kind information. For more details please the revise version of the paper and the attached code as well.

- Please compare the performance between various models and distinguish the results from existing models.

  Reply:-In this paper, we have performed experiments on large number of different type of images (hazy or foggy images captured under different homogeneous and inhomogeneous weather conditions like Outdoor and Indoor Scenes, Synthetic Road Scene Images Under Heterogeneous Fog, Realistic CCTV Camera Captured Images Under Heterogeneous Haze etc,) in order to validate the performance of the proposed model and its authenticity and to endorse the proposed model performance, we have included the famous Real-World Foggy Image Data-set (MRFID) as well. The performance of the proposed model is compared with other state-of-the-art models like(M1 and M2) qualitatively and quantitatively. For more details see the revised manuscript.

-) Please show the model dynamical training process, for example, the cost function of entropy should be decreased during the training. The following papers would be helpful: doi.org/10.1080/21642583.2019.1588804; doi.org/10.1016/j.neucom.2022.01.088; etc.

  Reply:- In this paper, four measures are taken to evaluate quantitatively the de-hazing outputs of the proposed model (M3) and its comparison with other recent existing methods (M1 and M2) in contrast and visibility recovery. These measures formulas or functions implementation dynamic training process have been shown in the paper and these have been taken from authentic published papers and literature. For instance, We have used MATLAB built in command/function/formula for measuring entropy of the output image. According to our information, a high entropy value means that the average amount of information in the image is high, which means there is a lot of information about the edges or features. Therefore, the higher the entropy value of the image, the better the contrast. The two dimensional information entropy of the image reflects the average amount of information in the image, the value range is [0 8]. The larger the value is, the more uniform the gray value of the image is. For more details, please see the following papers: https://doi.org/10.5573/IEIESPC.2017.6.2.085; DOI: 10.25165/j.ijabe.20181102.3357; doi:10.3390/s17122861; and MATLAB Built in commands etc. Hope our reply will be given due consideration and if there exists still any error/mistake/flaw, we will welcome your valuable comments for the improvement of the paper.

- The presentation and writing need to be improved. Previous works mean the works from the same author team. If not, it should be entitled as preliminaries.

  Reply:-An attempt has been made to improve further the presentation and writing of the manuscript. We have made necessary corrections according to the reviewer comments. For more details see the revised manuscript.

Second Reviewer:

- Manuscript is well-written and recent literature has been reviewed as well. The method seems to outperform some of the established models, especially produces better results in low-light/night settings and 'minimizes' halos.

  Reply: We are thankful for your encouraging remarks about our manuscript(work).

- My only concern is that the MATLAB code has not been made available, which would be great to review/verify the correctness of the code.

  Reply: We have provided the related pseudo MATLAB code of the proposed method for your kind information. For more details, please see the attached code.

- Figures are not labeled correctly, all figures miss the corresponding labels (a, b, c, ...).

  Reply: We have made necessary corrections according to your valuable comments. For more details, please see the revised manuscript.

We acknowledge the effort of the reviewers for giving us very useful suggestions.
The manuscript has been resubmitted to your journal. We look forward to your positive response.

Sincerely,

Dr. ASMAT ULLAH (corresponding author).
